# Profil des usagers de substances psychoactives: état des lieux après 13 mois d’activités du centre de soins « Colibri Sud »

**DOI:** 10.11604/pamj.2024.48.148.42102

**Published:** 2024-08-02

**Authors:** Cherileila Thiombiano, Charles Somé, Herman Bazié, Doriane Zombré, Sié Da, Adama Kantagba, Ollo Da, Abdoulaye Ouattara, Wilfried Wenceslas Bazié, Isidore Tiandiogo Traoré

**Affiliations:** 1 Centre Muraz, Institut National de Santé Publique, Bobo-Dioulasso, Burkina Faso,; 2 Institut Supérieur des Sciences de la Santé, Université Nazi Boni, Bobo-Dioulasso, Burkina Faso,; 3 Centre Médical Colibri Sud, Bobo-Dioulasso Burkina Faso

**Keywords:** Toxicomanie, profil, substances psychoactives, Addiction, profile, psychoactive substances

## Abstract

Pour améliorer l'accès aux soins des usagers de substances psychoactives dans la ville de Bobo-Dioulasso, le Centre de Soins, “Colibri Sud” a été créé. Cette étude avait pour objectif de décrire le profil des usagers de substances psychoactives qui ont fréquenté le Centre Médical “Colibri Sud” après 13 mois d'activités. Il s'est agi d'une étude transversale descriptive sur des données collectées du 1^er^ mars 2022 au 31 mars 2023. Les variables collectées étaient le sexe, l'âge, la profession et le type de substance consommée. L'analyse des données a été faite avec le logiciel STATA 16. Un test de Fisher exact de 5% a été considéré comme significatif. Au total, 116 patients ont été inclus dans notre étude. Les substances les plus consommées étaient le cannabis (75,86%), les benzodiazépines (25%), les opiacées (11,2%), les barbituriques 6,9%), cocaïne (3,44%). Les polyconsommateurs représentaient 15,50%. Les patients de moins de 30 ans étaient les plus représentés (67,24%) ainsi que les travailleurs du secteur informel (40,52%) et les élèves/étudiants (31,03%). Le centre est fréquenté par une population masculine à majorité jeune, le cannabis reste la substance la plus consommée. Cependant les benzodiazépines, les opiacées et la polyconsommation jadis rare est maintenant non négligeable. Au regard des profils retrouvés, l'accessibilité aux tests chromatographiques de détection et de quantification de ces substances psychoactives est plus que nécessaire pour une meilleure prise en charge. Aussi les actions entreprises doivent être surtout en faveur des élèves et étudiants.

The “Colibri Sud” care centre was set up to improve access to care for psychoactive substance users in the city of Bobo-Dioulasso. The purpose of this study was to describe the profile of psychoactive substance users who attended the “Colibri Sud” Medical Centre after 13 months of activity. We conducted a descriptive cross-sectional study based on data collected from March 1^st^, 2022, to March 31^st^, 2023. The variables collected included sex, age, occupation, and the type of substance used. Data analysis was performed using STATA 16 software. A Fisher's exact test with a 5% significance level was considered significant. A total of 116 patients were included in the study. The most commonly used substances were cannabis (75.86%), benzodiazepines (25%), opiates (11.2%), barbiturates (6.9%), and cocaine (3.44%). Poly-drug users accounted for 15.50% of the sample. Patients under 30 years of age were the most represented (67.24%), as well as workers in the informal sector (40.52%) and students (31.03%). The centre is frequented predominantly by a young male population and cannabis remains the most commonly used substance. However, the use of benzodiazepines, opiates, and poly-drug use, which was previously rare, is now significant. Given the profiles observed, access to chromatographic tests for detecting and quantifying these psychoactive substances is crucial for better management. Additionally, actions undertaken should particularly target students.

## Introduction

La toxicomanie se définit comme une perte de contrôle sur la consommation de substances psychoactive ou la recherche compulsive de ces substances malgré les conséquences néfastes [1]. La drogue ou substance psychoactive désigne l'ensemble des produits agissant sur le cerveau et peuvent entraîner des changements dans les perceptions, l'humeur, la conscience, le comportement et diverses fonctions psychologiques [2]. L'utilisation et l'abus de ces substances sont devenus de nos jours un problème majeur de santé des populations [3]. Elles peuvent entraîner chez les consommateurs des conséquences psychosociales telles que la dépendance et les troubles de santé mentale [4]. En 2020, le nombre de personnes âgées de 15 à 64 ans qui ont consommé la drogue au cours des 12 derniers mois dans le monde était estimé 284 millions, en majorité des hommes [5]. Le cannabis était la drogue la plus utilisée avec 209 millions d'utilisateurs en 2020, avec une augmentation de 23% du nombre de consommateurs de cannabis entre 2010 et 2020 [6]. Au Burkina Faso, la lutte contre la drogue tourne entre autre autour de quatre (03) axes, à savoir: la prévention, le traitement et la réinsertion sociale. Les stratégies de soins à adapter à l'endroit des victimes de drogues seront plus efficaces si les substances consommées sont clairement identifiées. Aussi, l'axe autour du traitement sera plus efficient si le profil des usagers des substances psychoactives est connu. En revanche peu de centres de soins sont dédiés à la prise en charge des usagers de substances psychoactives au Burkina. C'est pour combler ce gap que le Centre de Soins, d'Accompagnement et de Prévention des Addictions (CSAPA) Colibri Sud a été créé à Bobo-Dioulasso, la deuxième ville du Burkina Faso. Après 13 mois de fonctionnement, il est important de faire un état des lieux des activités, et d'identifier le profil des usagers de substances psychoactives reçues en consultation au centre. Cela pourrait améliorer les stratégies de prévention et de prise en charge, aider à anticiper les défis qui se poseront. C'est dans cette optique que cette étude a été initiée avec pour objectif général de décrire le profil des usagers de substances psychoactives qui ont fréquenté le Centre Médical « Colibri Sud » après 13 mois d'activités.

## Méthodes

**Cadre de l'étude:** cette étude a été réalisée au niveau du Centre de Soins, d'Accompagnement et de Prévention des Addictions (abus des drogues) (CSAPA) « Colibri Sud ». Ce centre médical affilié à l'association « Colibri Sud » a pour objectif de contribuer à la prévention et à la prise en charge de l'abus des substances psychoactives, des groupes vulnérables sur le plan sanitaire, psychosocial et économique dans la ville de Bobo-Dioulasso au Burkina Faso.

**Type et période d'étude:** il s'est agi d'une étude transversale à visé descriptive qui s'est déroulé du 1^er^ janvier 2023 au 30 mai 2023 sur des données collectées du 1^er^ mars 2022 au 31 mars 2023.

**Population d'étude**

***Critères d'inclusion:*** nous avons inclus dans notre étude tous les usagers de substances psychoactives reçus au Centre Médical « Colibri Sud » pour une consultation avec l'équipe médicale du 1^er^ mars 2022 au 31 mars 2023 avec un test rapide urinaire de dépistage positif et qui ont donné leur consentement éclairé pour participer à l'étude. Les substances détectables par le test étaient: les amphétamines, les barbituriques, les benzodiazépines, la cocaïne, le cannabis, les méthamphétamine et les opiacés. Le test utilisé était le Nova test du laboratoire Atlas Link Biotech (Chine), un test immunochromatographique.

***Collecte et analyse des données:*** la collecte des données a été faite à l'aide d'une fiche de collecte élaborée à cet effet. Les variables suivantes ont été collectées: le sexe, l'âge, la profession et le type de substances consommées. La saisie des données a été faite sur Excel 2016 et l'analyse des données a été faite avec le logiciel STATA 16. Nous avons procédé à une analyse descriptive des caractéristiques et substances consommées sous forme de moyenne pour les données quantitatives et de proportions pour les données qualitatives. Un test de Fisher exact inférieur à 5% a été considéré comme significatif.

**Considération éthique:** afin de garantir la confidentialité des données, tous les outils de collecte ont été dénominalisés. Le consentement des patients a été recueilli. Les données ont été enregistrées dans une base élaborée à cet effet et dont l'accès est limité et conditionné par l'utilisation d'un mot de passe. Les données ont été recueillies suivant les règles d'éthique et déontologique. Par conséquent, la confidentialité de chaque patient a été respectée.

## Résultats

Durant la période d'étude, 116 patients usagers de drogues ont bénéficié d'une consultation médicale avec test de dépistage urinaire positif aux drogues.

**Caractéristiques sociodémographiques:** l'âge moyen des patients était de 27,53 ± 9,64 ans avec des extrêmes de 15 ans et 65 ans. Les moins de 30 ans était les plus représenté (67,24%). Le sexe masculin était majoritaire (95,69%). Ceux du secteur informé et les élèves/étudiants majoritaire (Tableau 1).

**Tableau 1:** caractéristiques sociodémographiques

Caractéristiques	Effectif (n=116)	Proportion (%)
Sexe masculin	111	95,69
**Age (années)**		
Moyenne (écart type)	27,53 (9,64)	
< 30	78	67,24
30 ans et plus	38	32,76
**Profession**		
Commerçant	17	14,65
Elèves/Etudiants	36	31,03
Sans emplois	16	13,8
Secteur informel	47	40,52

**Figure 1:**
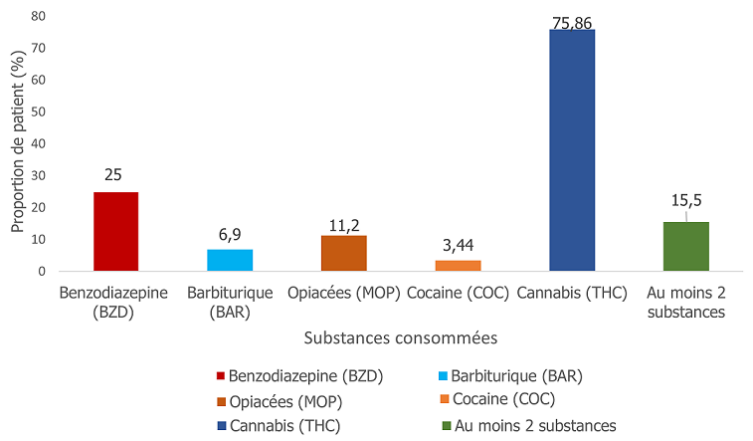
types de substances consommées par les patients fréquentant le centre de soins

**Type de substances consommées:** les substances les plus consommées étaient le cannabis (75,86%), suivie des benzodiazépines (25%), et les opiacés (11,2%). Les polyconsommateurs représentaient 15,50% (Figure 1).

**Substances consommées par tranches d'âges des patients:** la répartition de la consommation en fonction des tranches d'âge montre que le cannabis était retrouvé à tous âges (Tableau 2).

**Tableau 2:** consommation des substances psychoactives en fonction des tranches d’âges

Type de drogue % (n)						
Classe d'âge	BZD	BAR	MOP	THC	Au moins deux de ces substances	Total
< 30 ans	11,54% (9)	6,41% (5)	6,41% (5)	58,97% (46)	16,67% (13)	100% (78)
30 ans et plus	10,53% (4)	2,63% (1)	5,26% (2)	68,42% (26)	13,16% (5)	100% (38)
Total	11,21% (13)	5,17% (6)	6,03% (7)	62,07% (72)	15,52% (18)	100% (116)
**Fisher's exact =0.917**						

**Substances consommées selon la profession:** les sans-emplois étaient les plus retrouvés dans la polyconsommation (Tableau 3).

**Tableau 3:** substances consommées en fonction de la profession

Type de drogue % (n)												
Profession	BZD		BAR		MOP		THC		Au moins deux substances		Total	
Commerçant	5,88%	(1)	5,88%	(1)	11,76%	(2)	58,82%	(10)	17,65%	(3)	100%	(17)
Elève/Etudiant	5,56%	(2)	5,56%	(2)	11,11%	(4)	61,11%	(22)	16,67%	(6)	100%	(36)
Sans emploi	0%	(0)	6,25%	(1)	0%	(0)	68,75%	(11)	25%	(4)	100%	(16)
Secteur informel	21,28%	(10)	4,26%	(2)	2,13%	(1)	61,7%	(29)	10,64%	(5)	100%	(47)
Total	11,21%	(13)	5,17%	(6)	6,03%	(7)	62,07%	(72)	15,52%	(18)	100%	(116)
**Fisher's exact = 0.278**												

## Discussion

Pour combler un gap qui est un besoin en centres de soins dédiés à la prise en charge des usagers de substances psychoactives au Burkina Faso, le Centre de Soins, d'Accompagnement et de Prévention des Addictions (CSAPA) « Colibri Sud » a été créé en mars 2021. Après 13 mois d'activités, un état des lieux sur le profil des usagers de substances psychoactives fréquentant le centre a montré que le centre est fréquenté par une population jeune à majorité masculine. La substance la plus consommée reste le cannabis suivi des benzodiazépines et des opiacées. Cependant un pourcentage non négligeable de poly consommateur a été retrouvé. Les élèves/étudiants représentaient environ un tier (1/3) de notre population d'étude. La tranche d'âge la plus concernée dans notre étude était celle de moins de 30 ans soit 67,24%. Nos résultats montrent que la jeunesse est plus vulnérable et paye le lourd tribu de ce fléau. En effet les jeunes seraient plus facilement influencés par les paires comme le montre l'étude de N'Dri *et al*. en Côte d'Ivoire [7]. Le Burkina Faso étant en majorité composé de jeunes, il est alors urgent de trouver une solution pérenne à ce fléau.

La prédominance masculine était de 95,69% des patients. Cette prédominance masculine est contraire au sexe ratio présent dans la population générale du Burkina [8]. Mais comparable à celle retrouvé partout dans le monde [6]. Les jeunes adultes masculins sont les plus consommateurs de ces substances psychoactives. Le cannabis est la drogue la plus consommée dans notre population d'étude (62,07%). Ce même constant est retrouvé par le réseau ouest-africain d'épidémiologie sur la consommation de drogues (WENDU) qui montre que de 2016 à 2019 au Burkina Faso, le cannabis était la drogue la plus consommée chez les patients sous traitement de la dépendance aux drogues à des proportions respectives pour chaque année de 68,1%, 60,7%, 37,3%, 52,7% [9]. Aussi la même tendance a été retrouvée chez les patients admis au service de psychiatrie de l'hôpital Sourou Sanou en 2020 avec 75% des patients positifs au cannabis [10]. Les polyconsommateurs et les opiacées étant aussi retrouvés, montre la nécessité pour une meilleure prise en charge d'avoir des méthodes de détection et de quantification de référence de ces substances pour pouvoir identifier les molécules telle que l'héroïne afin de pouvoir envisager des traitements de substitution. Le secteur informel étaient majoritaires (40,52%), suivi des étudiants/élèves du (31,03%). Le réseau ouest-africain d'épidémiologie sur la consommation de drogues (WENDU) avait rapporté une proportion de 10% d'élèves/étudiants chez les personnes en traitement de la dépendance aux drogues au Burkina Faso en 2018 et 2019 [9]. La consommation en milieu scolaire semble prendre de l'ampleur et nécessite une attention particulière.

Parmi les élèves et étudiants, nous avons retrouvé que 72,22% prenaient du cannabis, suivi des benzodiazépines et des opiacées, mais aucune consommation de cocaïne n'a été retrouvé dans le groupe des élèves/étudiants. Cependant Nikiema *et al*. a trouvé dans son étude sur la consommation des psychotropes au Burkina Faso une faible consommation de cannabis en milieu scolaire, soit une proportion de 1,73% [11]. Le profil de consommation des substances psychoactive en milieu scolaire aurait probablement changé depuis 2011 au Burkina Faso et pourrait s'expliquer par une plus grande accessibilité au cannabis, comme en témoigne la quantité de cannabis saisi selon le Comité National de Lutte contre les Drogues en 2022. En effet plusieurs études ont montré que des facteurs environnementaux tel que la disponibilité de la substance, l'exposition sociale à la consommation pouvait influer sur l'usage problématique du cannabis [12,13].

## Conclusion

Treize mois après le fonctionnement du Centre de Soins, d'Accompagnement et de Prévention des Addictions (CSAPA) « Colibri Sud », 116 patients ont été consultés. Les substances les plus consommées étaient le cannabis. Cependant la consommation de benzodiazépine et des opiacées est aussi d'actualité une polyconsommation a aussi été constatée. Le secteur informel était majoritaire suivi des élèves/étudiants. Des enquêtes spécifiques auprès de la population générale sont indispensables pour mieux connaître le profil épidémiologique de ces consommateurs afin de mieux planifier une offre de soins adaptée. Aussi une étude des facteurs associés à cette consommation est nécessaire pour mieux comprendre ce fléau. Par ailleurs au regard des profils retrouvés, l'accessibilité aux tests chromatographiques de détection et de quantification de ces substances psychoactives est plus que nécessaire pour une meilleure prise en charge. Aussi les actions entreprises doivent être surtout en faveur des élèves et étudiants.

### Etat des connaissances sur le sujet

- *Le cannabis est reconnu comme la substance psychoactive la plus consommée dans le monde;*
- *Au Burkina Faso, il y a un manque de données récentes sur la toxicomanie; cependant les études déjà réalisées allaient dans le même sens avec une consommation en majorité de cannabis; il ne ressortait qu'une très faible consommation des autres substances psychoactives;*
- *La prise en charge ne nécessitait pas de traitement de substitution car les études au Burkina Faso ne montraient que de rare cas de consommation d'opioïdes tel que l'héroïne ou le tramadol.*

### Contribution de notre étude à la connaissance

- *Nous apportons des données récentes sur la consommation des substances psychoactives, pour pallier à un manque de données récentes sur la toxicomanie au Burkina Faso;*
- *Le cannabis est toujours la substance la plus consommée, il y a une augmentation de la consommation des autres substances tel que les benzodiazépine, les opiacées (tramadol, héroïne) et aussi une hausse de la polyconsommation;*
- *Les stratégies de prises en charge jadis adoptées au Burkina Faso devraient être revues avec d'abord, une mise à jour des méthodes de dosage en favorisant des méthodes de pointes qui pourrait identifier et quantifier la substance consommée, puis rendre disponible les médicaments de substitution, car les opiacées sont de plus en plus incriminés dans les addictions.*
